# Cholecystocutaneous fistula complicating percutaneous drainage of a pericholecystic abscess, managed conservatively

**DOI:** 10.1093/jscr/rjag799

**Published:** 2026-09-03

**Authors:** Joshua J Huggett, Chinedum Anosike, Frances E Oldfield, Noaman Sarfraz

**Affiliations:** Department of General Surgery, North Cheshire and Mersey NHS Foundation Trust, Warrington WA5 1QG, United Kingdom; Department of Radiology, North Cheshire and Mersey NHS Foundation Trust, Warrington WA5 1QG, United Kingdom; Department of General Surgery, North Cheshire and Mersey NHS Foundation Trust, Warrington WA5 1QG, United Kingdom; Department of General Surgery, North Cheshire and Mersey NHS Foundation Trust, Warrington WA5 1QG, United Kingdom

**Keywords:** cholecystocutaneous fistula, acute cholecystitis, pericholecystic abscess, conservative management, stoma appliance, biliary fistula

## Abstract

Cholecystocutaneous fistula (CCF) is a rare complication of gallbladder disease, in which an epithelialized tract forms between the gallbladder and skin. Its incidence has fallen with modern imaging, antibiotics and early cholecystectomy; an increasingly recognised cause is percutaneous gallbladder drainage. Definitive treatment is cholecystectomy with *en bloc* tract excision, but management of high-risk patients is poorly defined. We report a CCF arising 8 months after image-guided percutaneous drainage of a pericholecystic abscess in a 91-year-old nursing home resident with multiple comorbidities. Given high operative and anaesthetic risk, the fistula was managed conservatively with a stoma appliance and multidisciplinary support. At 3 months she remained well, with a matured, intermittently draining fistula and spontaneous gallstone extrusion. To our knowledge this is the first iatrogenic CCF reported in a nonagenarian, and the first with serial photographic documentation of its evolution, supporting conservative containment as a safe, patient-centred alternative to surgery.

## Introduction

A cholecystocutaneous fistula (CCF) is an abnormal epithelialized tract between the gallbladder and the skin [1]. First described by Thilesus in 1670, the condition typically arises from neglected calculous cholecystitis: progressive intraluminal pressure from cystic duct obstruction precipitates mural ischaemia, necrosis and extraperitoneal perforation [1, 2]. Its incidence has fallen markedly with modern imaging, antibiotics and timely cholecystectomy, with <100 contemporary cases reported [1, 3]. The right upper quadrant is the most common cutaneous opening, although umbilical, flank, gluteal and mammary sites have been described [1, 3]. An increasingly recognized aetiology is iatrogenic CCF following percutaneous cholecystostomy, whereby epithelialization along the drain tract creates a persistent biliary-cutaneous communication after drain removal [4, 5]. Definitive treatment remains open or laparoscopic cholecystectomy with *en bloc* excision of the fistulous tract, while conservative management is reserved for patients in whom surgery is prohibitive [1, 2, 6].

## Case report

A 91-year-old female nursing home resident presented to the surgical admissions unit of a busy district general hospital with a 6-week history of an enlarging, painful right upper quadrant (RUQ) mass, early satiety and malaise. In the weeks prior to admission, she had attended the emergency department following a fall, during which the mass was incidentally noted and referred for outpatient computed tomography (CT). Her past medical history included heart failure with reduced ejection fraction, atrial fibrillation, hypertension, osteoporosis and a previous basal ganglia infarct. Past surgical history consisted of an open appendicectomy as a young woman. Regular medications included apixaban, bisoprolol, ramipril, spironolactone, and omeprazole.

**Figure 1:**
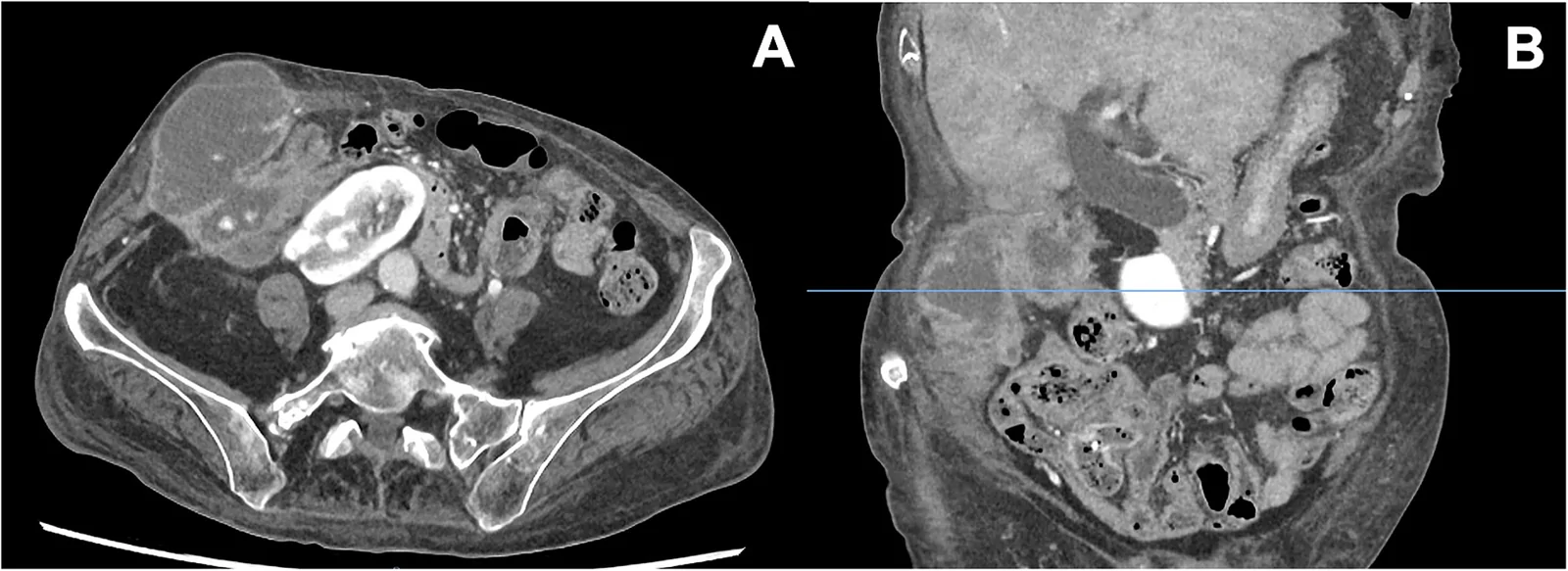
Axial (A) and coronal (B) views demonstrating a perforated gallbladder with a collection extending into the anterior abdominal wall with gallstones *in situ.*

Examination revealed a well-demarcated, firm, immobile, irreducible and tender 5 × 5 cm RUQ mass; the rest of the abdomen was unremarkable. She was apyrexial with observations within range. Bloods showed a white cell count of 19.5 × 10^9^/L, C-reactive protein (CRP) 126 mg/L and mildly deranged liver function tests. Differentials included an incarcerated abdominal wall hernia, given the irreducibility; abdominal wall abscess, in keeping with the inflammatory picture; and underlying malignancy, which warranted exclusion. Intravenous (IV) amoxicillin, metronidazole and gentamicin were commenced. Contrast-enhanced CT of the abdomen and pelvis demonstrated acute calculous cholecystitis with a perforated gallbladder and a contiguous pericholecystic abscess arising from the perforation, tracking into the right anterior abdominal wall (Fig. 1).

Given her comorbidities, ultrasound-guided percutaneous drainage with an 8 French pigtail catheter drained 100 ml of bile-stained pus before dislodging at 7 days. Drain fluid culture grew *Citrobacter braakii* and *Klebsiella pneumoniae*, sensitive to co-trimoxazole, which guided oral step-down therapy. Infection markers normalized and she was discharged on Day 11 on a 4-week course of oral co-trimoxazole and metronidazole, with weekly outpatient blood monitoring. The admission was complicated by a right proximal deep venous thrombosis, treated with 3 months of apixaban at an escalated dose.

**Figure 2:**
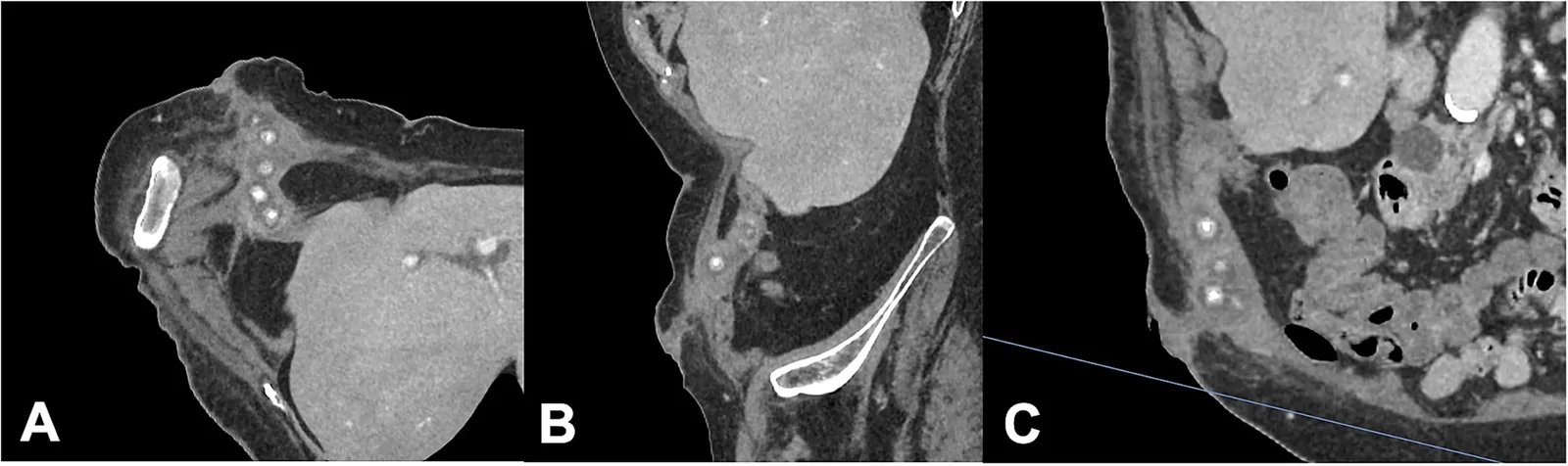
Axial (A), sagittal (B), and coronal (C) views of a large (max length 10.2 cm), irregular, wall-enhancing lesion abutting the inferior aspect of the liver and extending down to the level of the sacrum. The lesion tracks through the abdominal wall into cutaneous tissues representing a cholecystocutaneous fistula. There are six high-density foci within; consistent with gallstones retained in the fistulous tract.

Eight months later she re-presented with a 5-day history of a tender, erythematous swelling in the right hypochondrium discharging purulent yellow fluid. She was systemically well (temperature 36.4°C). Her history now included vascular dementia following a second ischaemic stroke; she mobilized with a walking frame, requiring one carer for all activities of daily living. Examination revealed a raised, tender 3 × 3 cm swelling with surrounding erythema; the abdomen was otherwise unremarkable. Bloods were within range apart from a CRP of 11 mg/L. CCF was the leading differential given the recent perforation and drainage; abdominal wall abscess was less likely biochemically. IV co-amoxiclav was commenced empirically. Contrast-enhanced CT confirmed a stone-containing fistulous tract extending from the RUQ through the anterolateral abdominal wall to the skin (Fig. 2). A wound swab yielded no growth.

Three management options were considered. Interval cholecystectomy with fistula tract excision, the definitive treatment in most reported cases [1, 3], was deemed to carry prohibitive perioperative risk due to left ventricular systolic dysfunction, advancing frailty and vascular cognitive impairment, with negligible quality of life benefit over conservative management. Repeat percutaneous drainage [4, 5] was not feasible on interventional radiology review. Conservative management with a stoma appliance was therefore selected. After agreement with the patient and family, the specialist stoma team fitted a stoma bag and educated the patient, family and nursing home staff.

**Figure 3:**
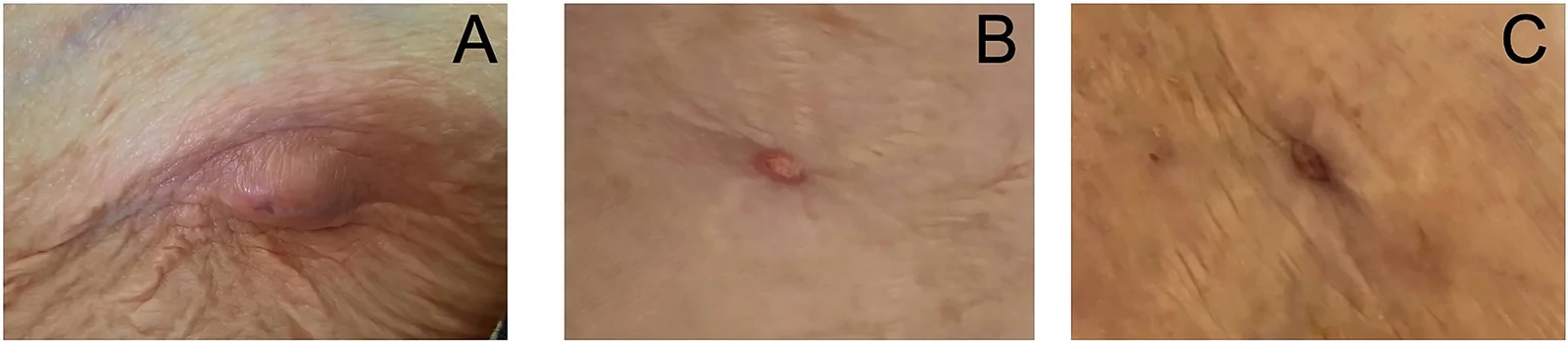
Serial clinical photographs of the cholecystocutaneous fistula. Day 1 of admission (A); 1 month following discharge (B); 3 months following discharge (C), showing the matured fistula tract.

At 3 months following discharge, she remained clinically well, with a soft, non-tender abdomen and no systemic features of infection. The fistula had matured (Fig. 3) and drained intermittently, with one episode of spontaneous gallstone extrusion, and she continued to manage the stoma appliance well.

## Discussion

CCF remains an extremely rare pathology. The classical presentation is a discharging right upper quadrant sinus on a background of long-standing or recently treated biliary disease, although diagnosis is often delayed by non-specific symptoms and a low index of suspicion [1, 7]. Cross-sectional imaging is key to diagnosis, with CT preferred over ultrasonography for delineating the tract, characterizing adjacent collections and identifying intra-fistula calculi [1, 3]. Cholecystectomy with *en bloc* excision of the fistulous tract remains the curative approach in fit patients [1, 3], but a growing minority of frail or comorbid patients are managed conservatively where operative risk outweighs the benefits of surgery [1, 4, 6].

Our case adds to the small but expanding literature on CCF as a late iatrogenic complication of percutaneous gallbladder drainage [4–6]. Notably, the fistula declared itself 8 months after drain dislodgement, emphasizing that the at-risk window extends well beyond the immediate post-procedure period and warrants patient counselling. To our knowledge, this is the first reported iatrogenic CCF in a nonagenarian and the first to document the fistula’s evolution with serial clinical photographs. A previous report described stoma-bag containment after surgical incision and drainage [6]; we demonstrate the same approach following radiologically-guided drainage at substantially greater age. CCF should remain on the differential in any patient with delayed cutaneous discharge following percutaneous gallbladder drainage. In carefully selected patients, where the risks of surgery outweigh any benefit, conservative containment with a stoma appliance, supported by specialist stoma input and clear, safety-netted follow-up, offers a legitimate, patient-centred alternative to surgery that prioritizes quality of life and dignity.

## References

[1] Brimo Alsaman MZ, Mazketly M, Ziadeh M et al. Cholecystocutaneous fistula incidence, etiology, clinical manifestations, diagnosis and treatment. A literature review. Ann Med Surg 2020;59:180–5. 10.1016/j.amsu.2020.09.035 PMC7554209 33082947

[2] Flora HS, Bhattacharya S. Spontaneous cholecystocutaneous fistula. HPB 2001;3:279–80. 10.1080/136518201753335584 18333032 PMC2020630

[3] Sah R, Rawal SB, Malla S et al. Cholecystocutaneous fistula after cholecystectomy. J Surg Case Rep 2024;2024:rjae617. 10.1093/jscr/rjae617 39372394 PMC11451475

[4] Pripotnev S, Petrakos A. Cholecystocutaneous fistula after percutaneous gallbladder drainage. Case Rep Gastroenterol 2014;8:119–22. 10.1159/000362360 24847193 PMC4025145

[5] Hariharan D, Lobo DN. Spontaneous extrusion of gallstones after percutaneous drainage. Ann R Coll Surg Engl 2017;99:e1–2. 10.1308/rcsann.2017.0015 PMC5450293 28071951

[6] Naim AJ, De Robles MS. Cholecystostoma: unusual evolution of a cholecystocutaneous fistula following percutaneous incision and drainage of perforated cholecystitis. J Surg Case Rep 2023;2023:rjad477. 10.1093/jscr/rjad477 37846417 PMC10577009

[7] Shariatmadari I, Rossi C, Krishna K. Mistaken identity: an unexpected case of spontaneous cholecystocutaneous fistula formation. BMJ Case Rep 2021;14:e234191. 10.1136/bcr-2019-234191 PMC7871220 33547115

